# Exploring the feasibility of a culturally tailored infant nutrition intervention: a qualitative study of perspectives from community facilitators and attendees in a pilot randomised controlled trial – Nurture Early for Optimal Nutrition (NEON) in East London

**DOI:** 10.1136/bmjph-2024-001358

**Published:** 2024-10-31

**Authors:** Logan Manikam, Priyanka Patil, Ummi Bello, Subarna Chakraborty, Sumire Fujita, Joanna Drazdzewska, Oyinlola Oyebode, Claire Llewellyn, Kelley Webbmartin, Carol Irish, Mfon Archibong, Jenny Gilmour, Phoebe Kalungi, Neha Batura, Rana Conway, Monica Lakhanpaul, Michelle Heys, the NEON Steering Team, Atul Singhal

**Affiliations:** 1 Research Department of Epidemiology and Public Health, University College London, London, UK; 2 Aceso Global Health Consultants Private Limited, Singapore; 3 Aceso Global Health Consultants Limited, London, UK; 4 Women and Children First, London, UK; 5 Wolfson Institute of Population Health, Queen Mary University of London Faculty of Medicine and Dentistry, London, UK; 6 University College London Research Department of Behavioural Science and Health, London, UK; 7 London Borough of Newham, London, UK; 8 Children’s Health 0-19 Service, London Borough of Newham, London, UK; 9 Mile End Hospital, London, UK; 10 Institute for Global Health, University College London, London, UK; 11 University College London Population Policy and Practice Research and Teaching Department, London, UK; 12 University College London Great Ormond Street Institute of Child Health, London, UK; 13 Collaborative Centre for Inclusion Health, London, UK; 14 University College London, London, UK

**Keywords:** Public Health, Community Health, Epidemiologic Research Design

## Abstract

**Introduction:**

Appropriate and healthy feeding practices can enhance a child’s health, prevent obesity and reduce chronic metabolic disease risks. Given the ethnic variations in feeding practices and metabolic risk, interventions must be community specific. Culturally tailored grassroots interventions targeting infant feeding can induce behavioural changes, mitigating chronic metabolic disease risks in later life.

The aim of this study was to explore participant feedback and inform intervention delivery methods within marginalised communities.

**Methods:**

A pilot three-arm cluster randomised controlled trial was conducted in the Tower Hamlets and Newham boroughs of London, involving community participatory learning and action groups. The study recruited 186 South Asian (Indian, Bangladeshi, Pakistani and Sri Lankan) mothers or carers of children aged 0–2 years. Participants in intervention arms were invited to either face to face or online intervention arms, facilitated by trained multilingual community facilitators (CFs), offering culturally informed discussions on child nutrition and care practices. The qualitative study was embedded within this trial, collecting feedback through interviews and focus groups. Thematic analysis was employed to identify key themes, focusing on intervention fidelity and acceptance.

**Results:**

Of the initial attendees, 42 (from the remaining 153 at the study’s conclusion) and 9 CFs offered feedback on the intervention’s delivery and suggestions for enhancing community-based interventions’ success. Key findings highlighted the need for a more flexible approach to boost participation and the significance of providing accessible, translated documents and resources. Participants expressed a strong preference for a hybrid model of intervention delivery, combining face-to-face and online sessions to accommodate diverse needs.

**Conclusion:**

Parenting interventions, particularly for new mothers, may engage more of the target population by adopting a hybrid design. This would provide attendees with the flexibility to select the delivery method, session timings and the option to participate at any stage of the intervention. The study underscores the importance of cultural tailoring and flexible delivery methods in enhancing participation and engagement in community-based health interventions.

**WHAT IS ALREADY KNOWN ON THIS TOPIC**

- Before this study, it was understood that the first 1000 days post conception are vital for a child’s development, with significant impacts from nutrition and dental health. Despite the UK’s adoption of WHO feeding guidelines, non-recommended complementary feeding practices persist among South Asian (SA) families due to bicultural issues and conflicting information. Community-based participatory interventions have been effective in low-income and middle-income countries, but are underused in the UK.

**WHAT THIS STUDY ADDS**

- This study highlights the feasibility and acceptability of a culturally tailored community Participatory Learning and Action (PLA) group intervention for SA families in East London. It shows that participants and facilitators value structured community interventions and suggests a preference for a hybrid delivery model. It also underscores the need for printed, translated materials for better engagement.

**HOW THIS STUDY MIGHT AFFECT RESEARCH, PRACTICE OR POLICY**

- The findings advocate for adopting hybrid models in parenting interventions to accommodate diverse needs and preferences. Enhanced facilitator training and simplified data collection tools are essential where similar interventions are being evaluated. This study could influence future community-based health promotion strategies and policies, emphasising cultural tailoring and flexible delivery methods to improve intervention outcomes.

## Introduction

 The initial 1000 days post conception are crucial for a child’s physical and mental development, with nutrition and dental health significantly influenced by birth weight and weight gain during infancy.^1^ Consequently, feeding practices during this period can have lifelong effects on a child’s growth and development.^2^ Health and age-appropriate feeding practices are essential for overall health, preventing childhood obesity and reducing the risk of chronic metabolic conditions.^2^

The South Asian (SA) population in the UK exhibits significant disparities and is among the most disadvantaged compared with other minority groups.^3^ Despite the adoption of the WHO’s Infant and Young Children Feeding Guidelines, non-recommended complementary feeding practices persist, particularly within SA families.^4 5^ This is attributed to bicultural issues, low levels of acculturation and conflicting information.^4^,^7^

Migrants from low-income and middle-income countries (LMICs) to high-income countries face an increased risk of metabolic disorders.^8^ Therefore, culturally tailored interventions targeting infant feeding practices are critical for improved population health.

Currently, few culturally tailored early-life interventions exist in the UK.^9^ The UK’s National Health Service (NHS) offers guidelines for optimal nutrition, care and dental hygiene practices for children, but these are not tailored to specific cultural practices, resulting in suboptimal uptake. Additionally, due to the NHS 10-year Forward Plan and the NHS Five Year Forward View, which aim to unburden the NHS by emphasising health promotion at the community level, there is a clear need for low-cost culturally sensitive interventions tailored to specific communities.^10^

Community-based participatory interventions could be an effective solution.^11^ Past research in LMICs found Participatory Learning and Action (PLA) group approaches to be cost-effective strategies for improving maternal and newborn health.^12^,^15^ Furthermore, this approach is also recommended by the WHO as an effective low-cost strategy for community mobilisation on maternal and newborn health.^16^ The PLA approach involves an iterative process where community facilitators (CFs) guide attendees through a four-stage cycle of identifying issues, designing solutions, implementing them and evaluating the results.^17^

The Nurture Early for Optimal Nutrition (NEON) programme, designed to enhance infant feeding, care and dental hygiene practices in SA minority groups, employs a community-based, participatory approach. The programme comprises two stages: NEON 1, a formative and feasibility study, and NEON 2, an intervention development and a pilot randomised control trial (RCT) in two London boroughs—Tower Hamlets (TH) and Newham (NH). Evaluations of such community-based interventions typically use mixed methods, including survey questionnaires, observational data and qualitative interviews.^18^

This qualitative study aims to explore participant feedback and inform intervention delivery methods within marginalised communities. The qualitative approach is particularly suited to understanding the acceptability and feasibility of the NEON community-based PLA group programme, as it provides in-depth insights into participant experiences and the contextual factors influencing intervention success. This method is advantageous for evaluating intervention programmes, especially within culturally specific populations, as it allows for a nuanced understanding of community needs and preferences. The study focuses on SA (Indian, Bangladeshi, Pakistani and Sri Lankan) mothers or carers of children aged 0–2 years living in THs and NH boroughs in London, offering culturally informed discussions on child nutrition and care practices facilitated by trained multilingual CFs.

## Methods

### Study design

This study is an observational, cross-sectional, qualitative study embedded within the NEON programme’s pilot RCT. The NEON intervention programme aimed to evaluate the feasibility, acceptability and preliminary effectiveness of a culturally tailored PLA group intervention for mothers and carers in multilingual SA communities. The programme’s primary objective was to measure children’s body mass index (BMI) z scores. The intervention spanned over 14 weeks, featuring eight biweekly sessions divided into four phases: learn, identify, implement and evaluate. The study employed a three-arm pilot feasibility cluster RCT design, comparing face-to-face PLA, online PLA and usual care groups.

Implemented in the inner-city boroughs of TH and NH in East London, the programme targeted 263 participants and achieved recruitment goals in both areas. Multilingual CFs delivered the sessions, supported by a research assistant who monitored feedback and ensured consistency. Quantitative analyses included descriptive summaries and BMI z score effects, while qualitative evaluations used thematic framework analysis of participant and facilitator feedback. Full details of the pilot feasibility RCT methodology can be found in Manikam L *et al* ‘Nurture Early for Optimal Nutrition (NEON) participatory learning and action women's groups to improve infant feeding and practices in South Asian infants: pilot randomised trial study protocol.’^19^

The qualitative component aimed to evaluate the acceptability, feasibility and fidelity of the intervention from the perspectives of participants and CFs. The qualitative study’s design, guided by implementation science frameworks, sought to provide in-depth insights into the community-based participatory intervention’s effectiveness and cultural tailoring.

### Study population

The target population for this study was SA groups living in London Boroughs of TH and NH. These boroughs are considered to be among the 5% most deprived in England. The boroughs were analysed in terms of ethnic make-up; in each borough, the six wards with the highest density of Asian population were selected based on census data.^19^ Participants included mothers or carers of children aged 0–2 years from Indian, Bangladeshi, Pakistani and Sri Lankan backgrounds.

### Participants

All attendees and CFs who participated in the pilot study were eligible for the qualitative study. The selection criteria for the qualitative interviews included participation in the intervention sessions and willingness to provide detailed feedback.

### Patient and public involvement (PPI)

PPI was integrated throughout the pilot randomised controlled trial, starting with focus group discussions involving community members and health professionals. These discussions informed the intervention development and delivery phases. This research was codesigned and delivered by SA CFs. They were involved in protocol development, developing and delivering the intervention; interpretation of findings into appropriate and attainable recommendations for practice; review and revision of draft academic papers; dissemination activities and development of plain language summaries. The pilot study findings were disseminated to all participants and facilitators via brochures and a dissemination conference held at the study’s conclusion. Volunteers from the study were invited to share their experiences and benefits at the conference, fostering a broader discussion among attendees.

### Data collection

Qualitative data were collected through:

1. *Facilitators’feedback*: the PLA group facilitator report form, encompassing both closed and open-ended queries on intervention delivery, participant engagement, fidelity and the facilitator’s session insights, was collected by the research assistant (RA) from the community facilitators (CFs) post each PLA session. Additionally, facilitators underwent interviews, either in-person or telephonically, with a researcher at the 6-month follow-up after the final PLA session. The RA provided the topic guides to research interns/independent observers who conducted the interviews.
2. *Participants’feedback*: after the final women’s group PLA session, attendees were invited to provide feedback via a digital questionnaire (in English), although with low response rates, leading to subsequent invitations for either in-person or telephonic interviews conducted by an independent observer (research intern). These feedback mechanisms aimed to identify areas of improvement and address issues pertaining to recruitment, retention, acceptability, fidelity, reach (diversity), measures and contamination, in line with the topic guides established in NEON 1.

### Analysis

All interviews were digitally audio recorded using a Dictaphone and transcribed for analysis using the five steps of Framework analysis by Braun and Clarke (online supplemental material 1).^20 21^ This study used NVivo V.12 Plus for a rigorous qualitative analysis of transcripts from CFs and attendees, with a validated coding framework applied to all transcripts. Independent text coding and data analysis by a research assistant and interns identified data-driven themes and concepts, with discrepancies resolved through NEON Steering team discussions. Thematic analysis identified patterns in feedback, using both inductive and deductive approaches in the data analysis process. Interviews, designed within implementation science frameworks, probed key factors such as adaptability, complexity and compatibility, facilitating the collection of contextual insights and stakeholder perspectives.

### Researcher characteristics

The NEON study involved a principal investigator (male) and a research assistant (female), both with backgrounds in public health, and the principal investigator is also a paediatrician. Their expertise in public health and paediatrics provided a solid foundation for the study, although neither had prior relationships with the CFs or participants. The facilitators (females) were recruited through local community networks, local councillors and children/community centres in TH and NH. These facilitators were trained to deliver the intervention as well as support with data collection, that is, feedback from participants and survey responses.

The research team consisted of individuals (RA, interns) responsible for data collection and analysis. They received training specific to the study’s aims and methodologies, ensuring consistency and reliability in the data gathered. The qualitative feedback collected from participants were cross-verified by facilitators after transcription to make sure the information was interpreted accurately. The researchers’ lack of prior involvement with the facilitators helped maintain objectivity, while their professional backgrounds ensured a comprehensive understanding of the public health context and the intervention’s goals.

### Methodological integrity

Techniques to enhance trustworthiness and credibility included triangulation and researcher reflexivity. Facilitators’ feedback on transcriptions and findings, contextual framing of results and consistent analytical processes ensured data integrity.

## Results

The researchers conducted 9 interviews with CFs (n%=90%; n=2 face to face and n=7 by phone) and 42 interviews with attendees (n%=29%; n=18 face-to-face and n=24 by phone), between February 2023 and April 2023. The CF’s interviews lasted from 20 to 40 min with a mean duration of 35 min, whereas the attendees’ interviews lasted for 10–20 min with a mean duration of 15 min.

From our analysis of feedback/interview data covering the key topics of interest, we identified various themes in both facilitators and attendee interviews stated below. These themes and subthemes are summarised in onlinesupplemental materials 2  3) and are elaborated in detail.

### CF interviews

From the interview data, we inductively and deductively identified and explored the following key themes in the facilitators’ feedback, which were further broken down to subthemes based on responses:

1. Poor attendance of attendees.
2. Enhancing attendee engagement.
3. Flexibility in session delivery and timings.
4. Tool kit.
5. Weather conditions.
6. Lost attendees.
7. Training delivered.
8. Support provided by RA.
9. Impact on personal growth and community.
10. Language and cultural adaptation.
11. Data collection challenges.
12. Health visitors support.
13. Intervention fidelity.

### Attendance and engagement

#### Poor/non-attendance of attendees (lack of study knowledge, weather conditions, lost interest, circumstantial unavailability)

The study noted an initial decline in participants, largely attributed to facilitators’ inadequate comprehension of the study’s objectives, impeding their ability to communicate its benefits effectively. This initial low turnout led to demotivation among CFs and researchers, impacting the pilot feasibility trial and causing some facilitators to resign (see below), but as sessions progressed, increased attendance was observed due to growing community interest and word-of-mouth recommendations.

#### CF dropout (demotivation, waste of effort)

The facilitators’ substantial efforts in participant recruitment and session preparation were compromised by non-attendance, causing demotivation and potential withdrawal, while a lack of understanding of the study’s design and objectives, coupled with the workload, led to attrition among some facilitators.

#### Enhancing attendee engagement (repeated phone calls, WhatsApp reminders, reiterating study benefits)

All facilitators stated that they contacted each attendee multiple times through phone calls and WhatsApp group messages, reminding them of session timings and repeating study benefits to increase attendance and engagement.

#### Flexibility in sessions

Attendees’ dissatisfaction with the session structure led to advocacy for a hybrid delivery model, while facilitators’ flexibility in adjusting session timings was crucial for caregivers managing multiple responsibilities.

#### Weather conditions

**Figure 1:**
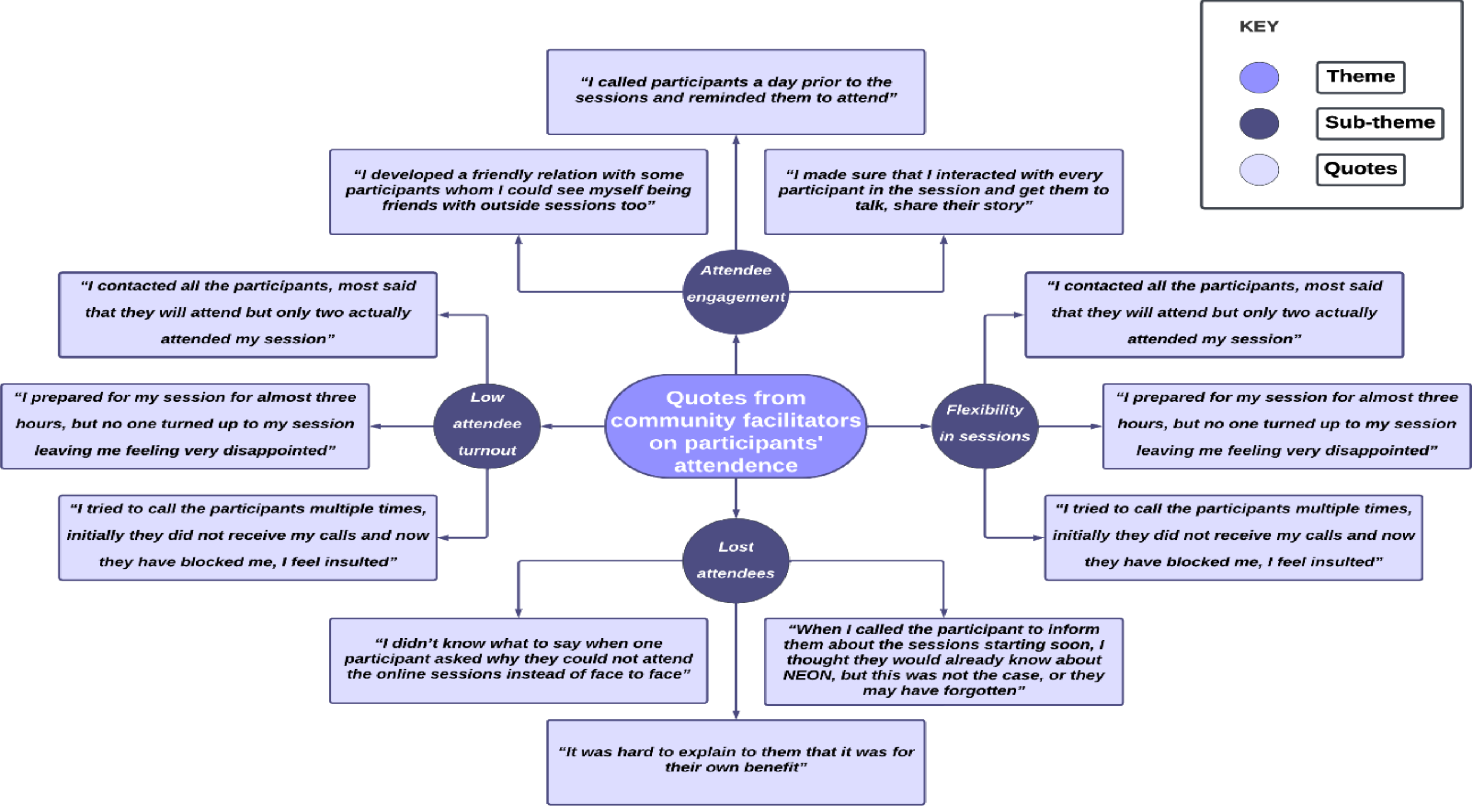
Quotes from community facilitators on participants’ attendance.

Adverse weather conditions, health-related issues among attendees or their children and changes in personal circumstances such as job commencement or relocation further contributed to irregular session attendance and to a preference for hybrid sessions (figure 1).

### Intervention delivery and fidelity

Hybrid model (flexibility in session delivery and timings (prayer time, etc)).

This was a frequent theme in most facilitator interviews, specifying the need to have flexibility in sessions and the opportunity to attend either of the online or face-to-face sessions provided.

#### Tool kits (picture cards, translations, updated versions, recipe books)

Most facilitators, especially those from TH, recommended translating session picture cards into local languages for improved comprehension and suggested revisions to the asset map and resource list to include additional resources, given the cost-of-living crisis. While the recipe book was unanimously deemed the most effective tool for session delivery, adherence to the manual on how to conduct the sessions, proved challenging for some facilitators, leading them to employ their preferred methods.

#### Training delivered (helpful, more sessions needed, involvement)

Many facilitators appreciated the training provided but thought it was rushed over 3 days and should have been broken down further with more hands-on activities and role plays to boost confidence and preparedness.

#### Intervention fidelity (delivered as proposed but this was the problem)

While most facilitators adhered to the manual and delivered the sessions as required, they believed that offering flexibility in timing, duration, and mode of attendance could have enhanced the benefits for the attendees.

#### Support provided by RA (helpful, team building)

Facilitators said the RA provided intermittent catchups which helped them share their experiences from sessions with each other, learn from each other, share information, motivate them and gave them a sense of working in a team.

Most facilitators said that it was good to have a single point of contact with whom they could discuss any ongoing issues. They suggested their biweekly meetings with the RA helped in team building and acted as a platform for them to share their thoughts on the sessions and challenges faced.

#### Impact on personal growth and community

The facilitators’ involvement in the programme, characterised by personal growth and significant community impact through infant nutrition education, is anticipated to result in lasting benefits such as improved child health and parenting skills, highlighting the programme’s multifaceted contributions.

Through the adaptation of materials to local languages and cultures, facilitators not only enhanced cultural understanding and disseminated knowledge on infant nutrition, thereby boosting self-confidence, but also contributed to education, facilitator empowerment and community development, with anticipated long-term benefits including improved child health and parenting skills.

#### Language and cultural adaptation

Facilitators, transcending mere knowledge transmission, fostered personal and community development through local language and cultural adaptations, thereby engendering participant trust and honouring cultural diversity, and despite the complexities in conveying nutritional concepts, they employed culturally relevant analogies, enhancing their cultural proficiency and making information more comprehensible for participants with limited English proficiency.

#### Support provided by health visitors (HV), baseline data collection challenges in NH

**Figure 2:**
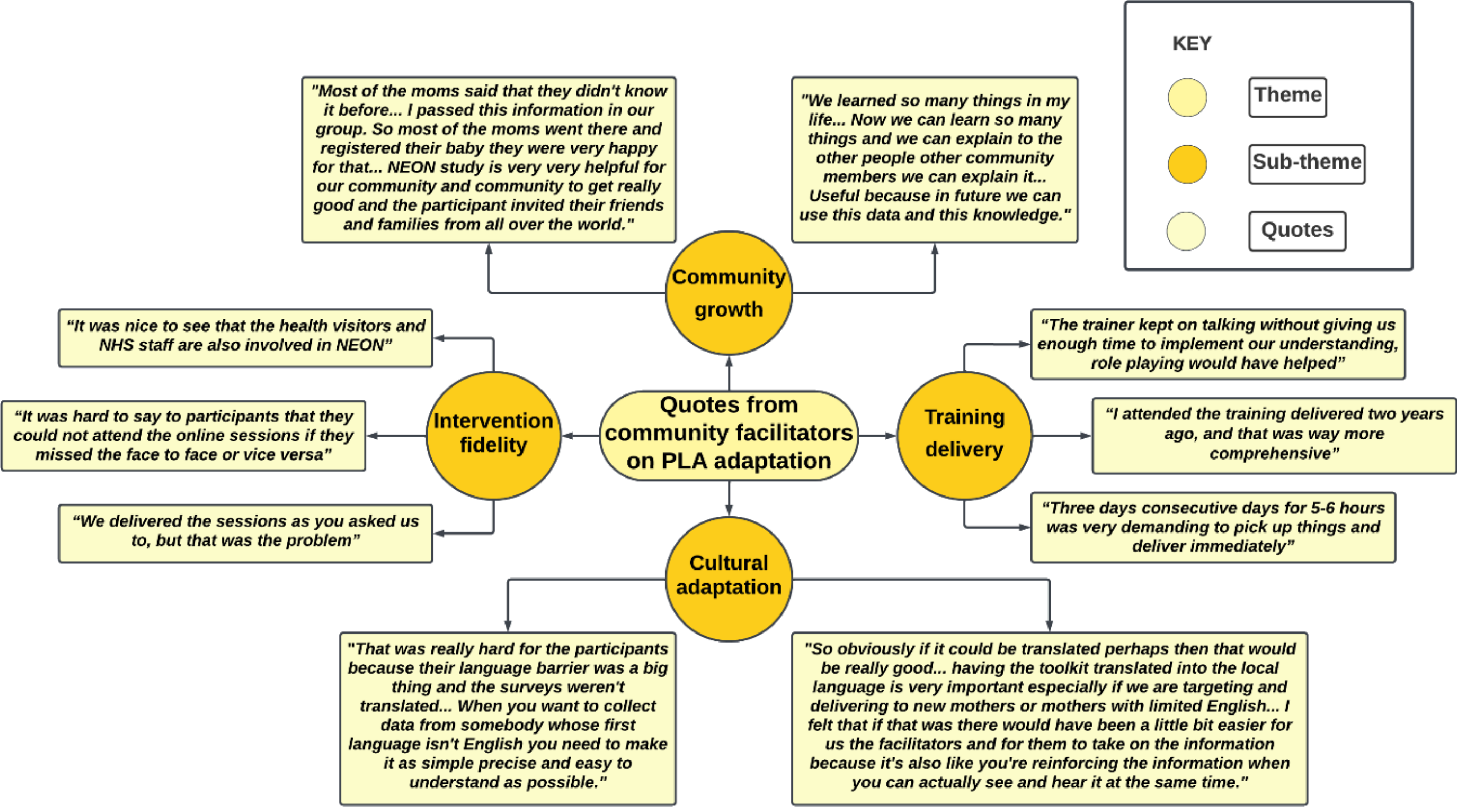
Quotes from community facilitators on language and cultural adaptation, training, intervention fidelity and coproduction. PLA, Participatory Learning and Action.

In TH, facilitators valued the assistance from HVs and children’s centres for initial data gathering on height and weight, yet encountered difficulties at the second data collection stage due to the requirement for attendees to schedule their own appointments instead of direct outreach from health visitors. Conversely, NH, despite not offering support for the baseline due to time restrictions, effectively handled data collection for the subsequent two timepoints without complications (figure 2).

## Data collection (very stressful, useless, poor response)

**Figure 3:**
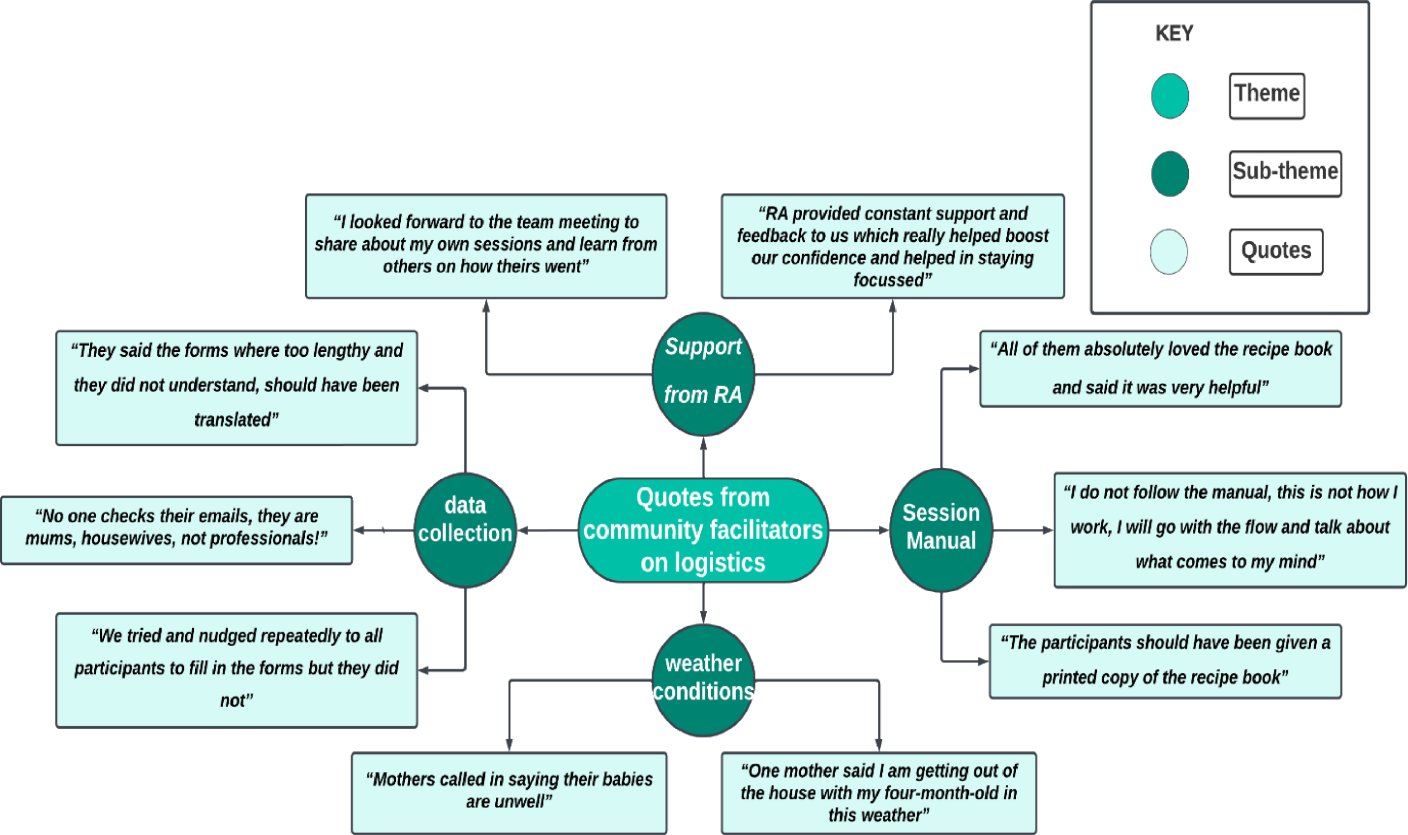
Quotes from community facilitators on logistics and support provided by Research Assistant RA).

Facilitators unanimously reported that the survey’s completion was impeded by the use of email links, absence of language translation and lack of incentives, resulting in a poor response rate despite repeated follow-ups. Facilitators identified the email distribution of questionnaires as a significant barrier due to its user-unfriendliness, proposed translations for improved accessibility and noted that surveys completed with CFs’ assistance resulted in the highest completion rates due to time efficiency and expert guidance (figure 3).

## Attendees’ interviews

In the thematic analysis of interview data, we identified the following key themes in the attendees’ feedback, which are summarised in online supplemental material 3.

1. The recruitment process.
2. Study explanation.
3. Non-participation/attendance.
4. Language barrier—picture cards and questionnaire.
5. Attitudes to the study.
6. Wider reach and inclusion.
7. Flexibility in sessions.
8. Session benefits.

## Recruitment

Most interested participants were referred by their HVs. However, alterations in personal circumstances impeded some from continuing, and those who became pregnant during the study were excluded due to the study’s inclusion/exclusion criteria.

## Study explanation

While most participants, introduced to the study via HVs, comprehended its objectives, they expressed confusion about its design, feeling deprived of choice. Conversely, participants in TH reported higher recruitment satisfaction, largely attributed to the engagement of advocacy assistants fluent in their native languages (Bangladeshi and Sylheti), enhancing their comfort levels.

## Non-participation/attendance

During the study’s initial stage, attendee numbers significantly dropped due to CFs’ contact regarding programme details, personal circumstance changes and feelings of exclusion, particularly among control arm participants.

## Language barrier

**Figure 4:**
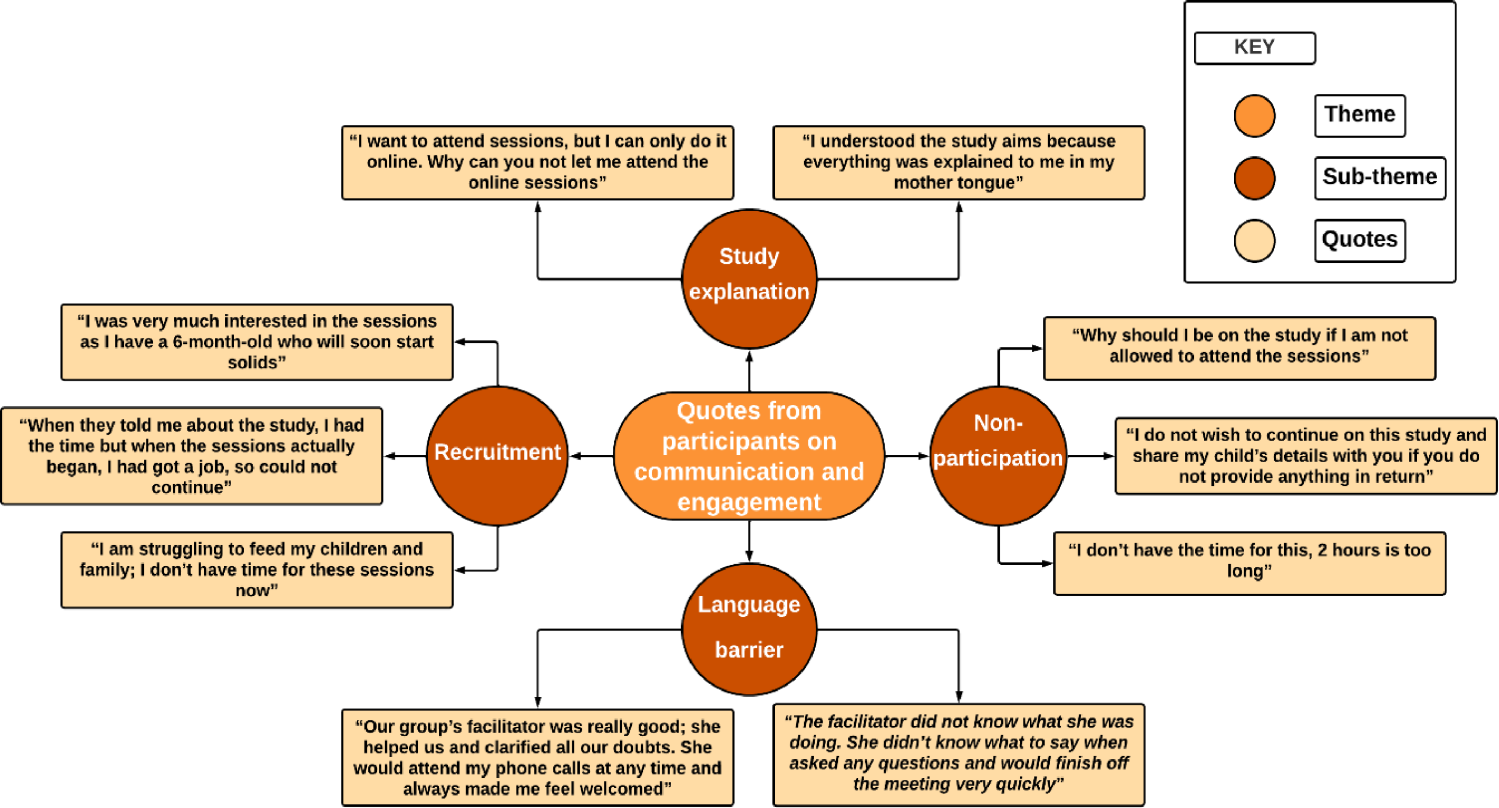
Quotes from participants on language barriers, understanding, recruitment and non-participation affecting communication and engagement.

Most attendees from TH expressed that they would have benefited significantly if the materials shared had been translated into their native languages. However, in NH, the response was more varied, indicating differences in English literacy levels within the two boroughs (figure 4).

### Attitudes to the study

In our study, ‘attitude’ was delineated as any belief, positive or negative, regarding the programme’s effectiveness, feasibility and the perception of the CFs. Our findings indicated that positive attitudes among CFs bolstered attendee commitment and acceptance of the programme, while a lack of programme awareness and negative CF attitudes adversely impacted attendee trust.

### Wider reach and inclusion

Regular attendees expressed interest in involving peers with children of similar age, attributing this to the sessions’ informative content and suggested expanding the programme to pregnant women. Parents of children aged 1–2 years regretted their late access to this information, believing it would have influenced initial feeding practices, and recommended introducing these sessions to pregnant women and new mothers to promote early beneficial habits.

### Flexibility in sessions

Almost all attendees suggested the need for having flexibility in the sessions and allowing them to choose how they wanted to attend these sessions—face to face or online.

### Benefits of the sessions

**Figure 5:**
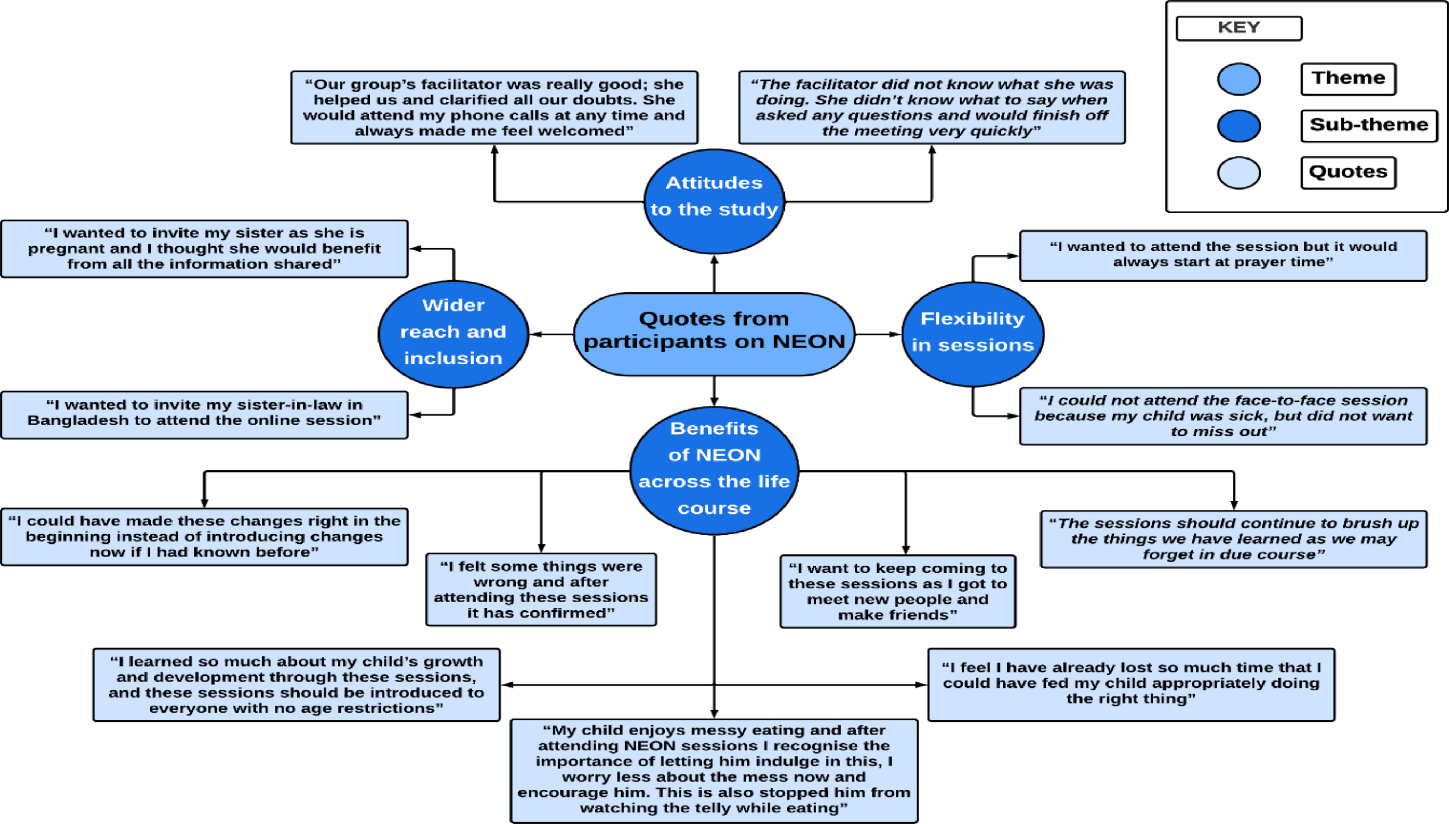
Quotes from participants on Nurture Early for Optimal Nutrition (NEON) intervention.

Participants who attended all eight sessions reported significant learning, which prevented the continuation of suboptimal practices. They expressed a desire for the continuation of these informative and enjoyable sessions, which also fostered a sense of camaraderie and comfort within the group. Participants who attended face-to-face sessions developed stronger relationships with the CFs and felt better supported (figure 5).

## Discussion

This study distinctively evaluates the feasibility of a culturally tailored community PLA group intervention through qualitative data to inform future implementation. Participants and facilitators recognised the significance of these structured community interventions in promoting community transformation, advocating for broader initiatives to include larger demographic groups and communities for optimal impact.

### Facilitator training and engagement

The facilitators’ comprehension of the study design and objectives was crucial to their engagement. Facilitators who had difficulty understanding the study’s aims and design, despite training, were less successful in representing the study to attendees and addressing their queries. This resulted in subpar session quality, leading to attendee attrition and subsequent facilitator disengagement. This finding aligns with previous studies that emphasise the importance of thorough and continuous training for facilitators to ensure high-quality delivery and engagement in community-based interventions.^22 23^

### Mode of delivery and intervention fidelity

Both facilitators and attendees expressed a strong preference for a hybrid delivery model, which was not fully realised in this study. Despite the pilot feasibility RCT incorporating multiple delivery modes, the lack of choice led to early study attrition. This supports findings from other studies that suggest flexibility in delivery modes can enhance participant retention and engagement.^24^ The rigidity in the current model (which was to assess the feasibility and acceptability of the delivery modes) underscores the need for flexibility to accommodate participants’ varying schedules and preferences.

### Resource utilisation and data collection

Both facilitators and attendees found the resources shared during the study sessions highly beneficial. However, most attendees preferred printed and translated versions of the resources, especially the recipe book, over digital copies. This finding highlights the importance of inclusive strategies that cater to specific populations with language barriers, echoing similar conclusions from other studies on community interventions.^25^ The preference for tangible materials underscores the need for culturally and contextually appropriate resources to enhance comprehension and engagement.

### Limitations

The study acknowledges potential limitation such as:

1. *Social desirability and respondent bias*: interviews conducted by CFs may have been influenced by social desirability, leading participants to provide more favourable responses.
2. *Language and translation issues*: some data may have been lost in translation, as interviews were not conducted in the primary researcher’s native language. This could affect the accuracy and depth of the qualitative data, potentially leading to misinterpretations or incomplete data capture.
3. *Scope of transferability*: the findings may not be fully transferable to other contexts or populations, given the specific cultural and demographic characteristics of the study sample.

Future studies should consider these limitations and employ strategies to mitigate their impact, such as using independent interviewers, ensuring accurate translations and conducting pilot tests to refine data collection methods.

## Conclusion

Parenting interventions, particularly those targeting new parents, could adopt a hybrid design to provide attendees with the flexibility to select the delivery method, session timings and participation stages that best fit their needs. This study suggests a hybrid delivery model, acknowledging the post-COVID-19 landscape where digital platforms offer significant benefits, but face-to-face sessions remain crucial for fostering community ties. Enhanced training for CFs is essential to ensure they fully understand the study design and objectives, enabling them to effectively engage with attendees. Simplified data collection tools and tailored designs are also recommended to enhance the feasibility and acceptability of community-based interventions.

## supplementary material

10.1136/bmjph-2024-001358 online supplemental file 1

10.1136/bmjph-2024-001358 online supplemental file 2

10.1136/bmjph-2024-001358 online supplemental file 3

## Data Availability

Data are available on reasonable request.
