# Supplementary figures and images for: Exploring the feasibility of a culturally tailored infant nutrition intervention: a qualitative study of perspectives from community facilitators and attendees in a pilot randomised controlled trial – Nurture Early for Optimal Nutrition (NEON) in East London
Source: BMJ Public Health. 2024 Oct 31;2(2):e001358. doi: 10.1136/bmjph-2024-001358 (PMC11816512; doi:10.1136/bmjph-2024-001358)

## Supplementary Material 1: Braun and Clarke's Framework Analysis

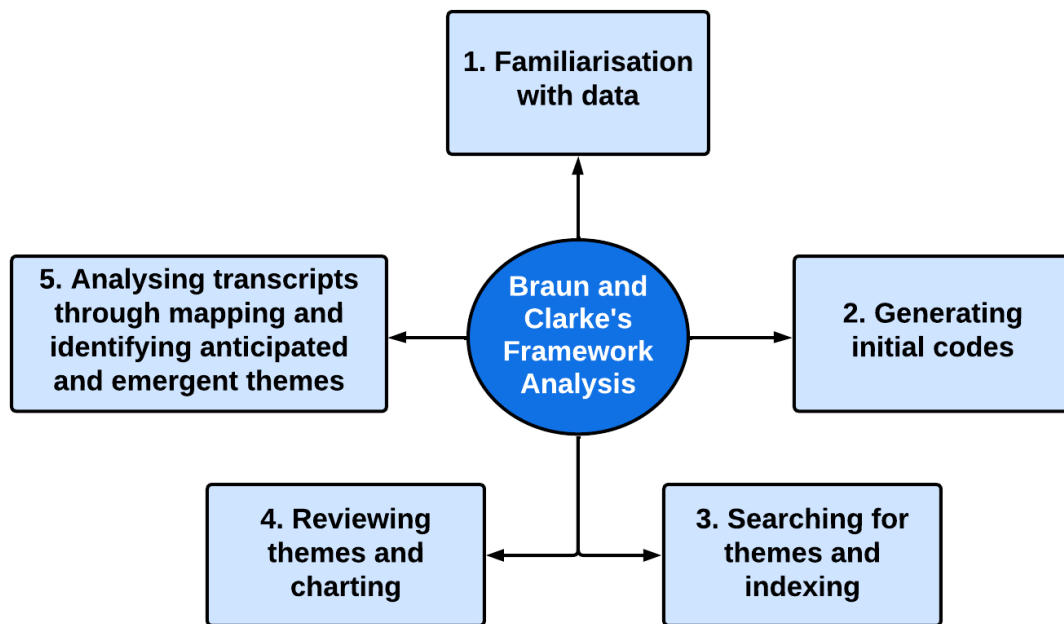

Supplement: online supplemental file 1 [file bmjph-2-2-s001.pdf]
